# Expanding the Reactivity of Masked Divalent Lanthanide–Isocarbonyl Complexes

**DOI:** 10.1021/acs.organomet.6c00042

**Published:** 2026-03-31

**Authors:** Siobhan R. Temple, Arpan Mondal, Sean R. Giblin, Jinkui Tang, Richard A. Layfield

**Affiliations:** † Department of Chemistry, School of Life Sciences, 1948 University of Sussex, Brighton BN1 9RH, U.K.; ‡ School of Physics and Astronomy, 2112 Cardiff University, Cardiff CF24 3AA, U.K.; § School of Chemistry and Chemical Engineering, 47833 Beijing Institute of Technology, Beijing 102488, P. R. China

## Abstract

Masked divalent reactivity is a potentially powerful strategy for accessing the reducing chemistry of lanthanides without the need to isolate unstable divalent lanthanide compounds. Here, we report the synthesis and characterization of the isocarbonyl-bridged complexes [(Cp*)_2_Gd­(THF)­(μ-Fp)]_∞_ (**1**_**Gd**_) and [(Cp*)_2_Lu­(μ-Fp)]_2_ (**1**_**Lu**_) (Fp = CpFe­(CO)_2_), aiming to extend the masked divalent approach to gadolinium and lutetium, metals with very limited divalent chemistry. Structural studies show that **1**_**Gd**_ forms as a coordination polymer with nine-coordinate gadolinium centers, whereas **1**_**Lu**_ is a dimer with eight-coordinate lutetium, reflecting the influence of the lanthanide contraction. FTIR spectroscopy confirms strong isocarbonyl back-bonding in both compounds. Complexes **1**_**M**_ (M = Gd, Dy, Lu) react with phenazine to afford dimetallic phenazine-bridged products [{(Cp*)_2_M}_2_(μ-phnz)] (**2**_**M**_), demonstrating two-electron reduction of an *N*-heterocyclic substrate by the isocarbonyl-bridged complexes. Magnetic measurements reveal extremely weak antiferromagnetic exchange in **1**_**Gd**_ and **2**_**Gd**_, while the dysprosium analogue exhibits slow magnetic relaxation governed by Raman and quantum tunnelling processes. These results establish isocarbonyl-bridged lanthanide metallocenes as platforms for ligand-based reduction chemistry involving metals with very limited divalent chemistry.

## Graphical abstract

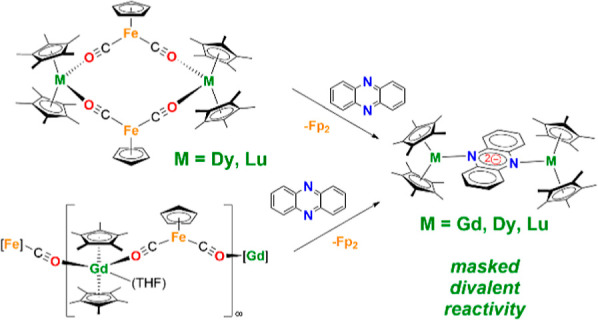

## Introduction

The chemistry of the rare-earth elements is dominated by the stable trivalent oxidation state, applications of which range from luminescent and magnetic materials to medical imaging and sensing.^1–3^ The divalent oxidation state is uncommon in rare-earth chemistry and has only been developed extensively for samarium, europium and ytterbium, whereas, for the other members of the series, molecular compounds containing divalent rare-earth elements are more recent developments. The instability of the divalent oxidation state can make it challenging to isolate compounds of these exotic species, thereby impeding the study of their chemistry. Despite the drawbacks, remarkable advances have been made, and properly characterized divalent compounds are now known for all rare-earth elements except promethium.^4–7^ Many significant developments have been pioneered using cyclopentadienyl ligands, including ‘ate complexes of the type [(η^5^-Cp^R^)_3_M]^−^ (Cp^R^ = C_5_H_4_SiMe_3_, 1,3-(Me_3_Si)_2_C_5_H_3_)^8–11^ and neutral metallocenes of the type [(η^5^-Cp^R1^)­M­(η^5^-Cp^R2^)] (Cp^R1^ = C_5_^*i*^Pr_5_, Cp^R2^ = C_5_^*i*^Pr_5_, C_5_Me_5_), some of which show single-molecule magnet (SMM) or spin qubit properties.^12–16^ Other ligands have also been used to stabilize divalent lanthanides, including bulky amide, amidinate, aryloxide and siloxide.^17–21^ Beyond characterization of the molecular and electronic structures of divalent rare-earth compounds, reactivity studies are less prevalent. However, the potent one-electron reducing character of nonclassical divalent lanthanides highlights considerable potential for development, particularly toward small-molecule activation.^22,23^

An alternative, indirect route into the chemistry of divalent rare-earth elements that circumvents the need to prepare potentially unstable compounds is to store the reducing electrons on ligands that are readily oxidized. Sterically induced reduction provides an example of this concept and is typified by the ability of bulky complexes such as (η^5^-Cp*)_3_M (Cp* = C_5_Me_5_), to react with mildly oxidizing substrates and transfer the redox-inactive trivalent lanthanide to the reduced substrate.^24,25^ The idea of ligand-based reduction chemistry has been pursued more widely in f-element chemistry, including, for example, ytterbium­(I) and uranium­(I) synthons that potentially shed light on the reactivity of this elusive oxidation state.^26–28^

We have recently reported trivalent rare-earth complexes of the end-on dinitrogen ligand [N_2_]^2–^ with the general formula [(η^5^-Cp^ttt^)_2_M­(μ-1,2-N_2_)] (M = Y, Gd, Dy; Cp^ttt^ = 1,2,4-^t^Bu_3_C_5_H_2_), which react as “masked” divalent reducing agents toward a variety of unsaturated organic substrates, eliminating N_2_ as a traceless leaving group.^29,30^ Comparable reactivity has also been discovered for other rare-earth dinitrogen complexes, including the photoswitchable lutetium metallocene [{(η^5^-C_5_Me_4_H)_2_Lu­(THF)}_2_(μ-1,2-N_2_)].^31,32^ The isocarbonyl-bridged trivalent rare-earth compounds [(Cp^ttt^)_2_M­(μ-Fp)]_2_ (M = Y, Dy; Fp = CpFe­(CO)_2_) have also been found to show masked divalent reactivity toward *N*-heterocycles, with the reducing electrons originating from the [μ-Fp]^−^ anions, which then combine to eliminate the classical dimer Fp_2_.^33^

To expand the masked divalent reactivity of isocarbonyl-bridged rare-earth compounds to metals with a wider range of ionic radii, we now report the gadolinium coordination polymer [(Cp*)_2_Gd­(THF)­(μ-Fp)]_∞_ (**1**_**Gd**_) and the lutetium dimer [(Cp*)_2_Lu­(μ-Fp)]_2_ (**1**_**Lu**_), noting that both metals have very limited chemistry in the divalent oxidation state.^9^ The reactivity of **1**_**Gd**_ and **1**_**Lu**_, along with that of previously reported [(Cp*)_2_Dy­(μ-Fp)]_2_ (**1**_**Dy**_),^34^ as reducing agents toward the benchmark substrate phenazine is also described, in addition to the magnetic properties of the ensuing gadolinium and dysprosium complexes of the phenazine dianion.

## Results and Discussion

The new isocarbonyl-bridged compounds **1**_**M**_ (M = Gd and Lu) were synthesized in THF using salt metathesis reactions between [(Cp*)_2_M­(BPh_4_)] and KFp, as described previously for **1**_**Dy**_ (Scheme 1).^34^ Both compounds were isolated as orange crystals, with yields of 43% and 50% for **1**_**Gd**_ and **1**_**Lu**_, respectively.

**Scheme 1:**
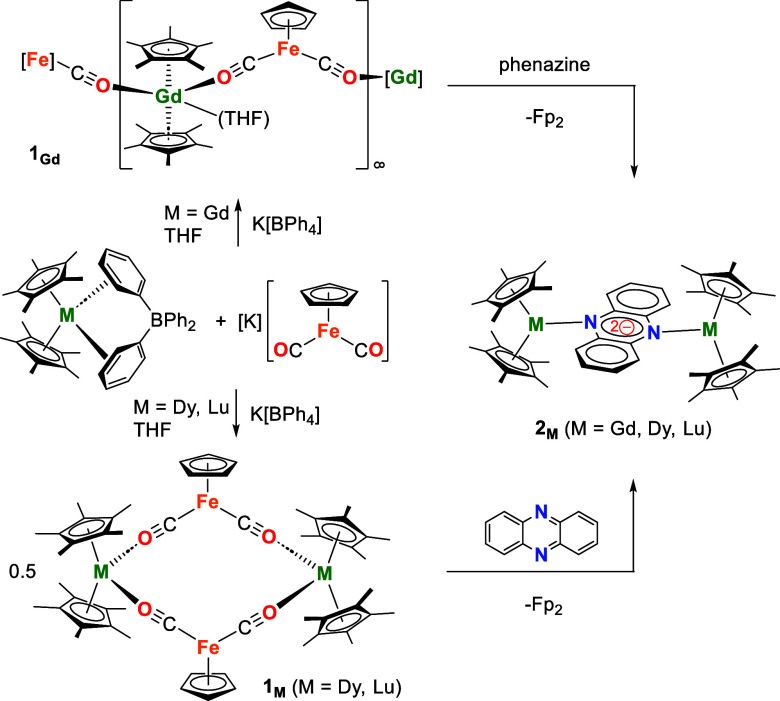
Synthesis of **1**_**M**_ and **2**_**M**_ (M = Gd, Dy, Lu)

The molecular structures of **1**_**Gd**_ and **1**_**Lu**_ were determined using X-ray crystallography (Figures 1 and 2, Tables S1 and S2). Compound **1**_**Gd**_ is a coordination polymer based on an asymmetric unit consisting of two bent {Cp*_2_Gd} metallocenes bound to a THF ligand and to the isocarbonyl oxygen atoms of neighboring [Fp]^−^ metalloligands, resulting in formally 9-coordinate gadolinium centers. The Gd–Cp* distances are in the range 2.4308(4)-2.4449(4) Å and the Cp*-Gd-Cp* angles are 136.78(9)° and 138.33(1)° for Gd1 and Gd2, respectively. The Gd–O distances to the isocarbonyl ligands are 2.410(4)-2.488(4) Å, and the associated O–Gd–O angles are 147.54(13)° and 147.76(13)° for Gd1 and Gd2, respectively. The shortest intrachain Gd···Fe separation is 5.2800(9) Å, and the intrachain Fe···Fe and Gd···Gd distances are 10.3209(13) Å and 8.4490(5) Å, respectively.

**Figure 1:**
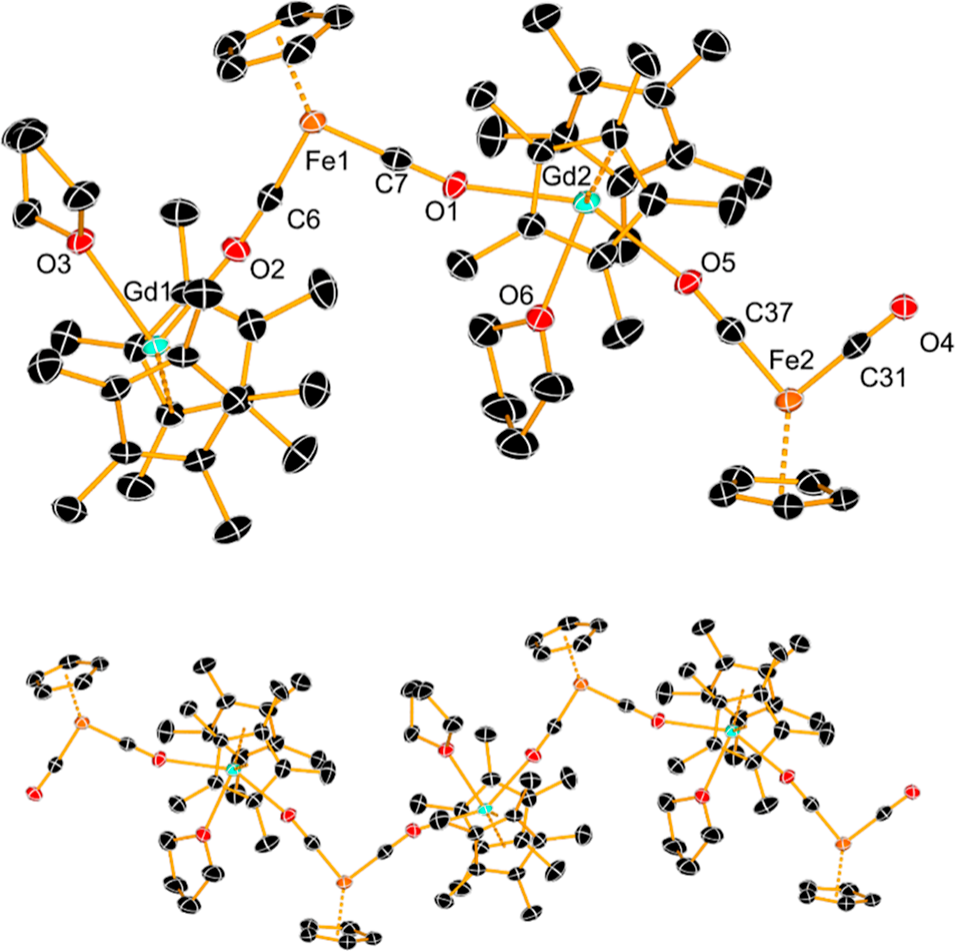
(Upper): asymmetric unit of **1**_**Gd**_. (Lower): extended segment of the coordination polymer structure. Hydrogen atoms are omitted for clarity, and thermal ellipsoids are set to 30% probability.

**Figure 2:**
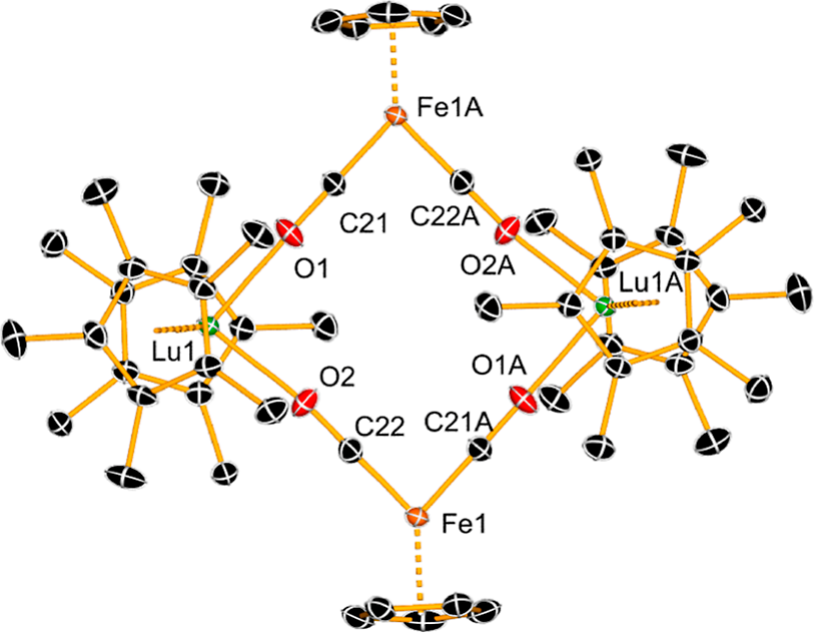
Molecular structure of **1**_**Lu**_. Hydrogen atoms are omitted for clarity, and thermal ellipsoids are set to 30% probability.

In contrast to **1**_**Gd**_, the lutetium isocarbonyl complex **1**_**Lu**_ is a centrosymmetric dimer with formally 8-coordinate lutetium centers, isostructural with the dysprosium analogue **1**_**Dy**_ (Figure 2). The Lu–(Cp*)_cent_ distances are 2.2816(18) Å and 2.2822(19) Å and the Cp*–Lu–Cp* angle is 141.70(6)°. The Lu–O distances in **1**_**Lu**_ are 2.220(3) Å and 2.228(3) Å, both markedly shorter than the analogous distances in **1**_**Gd**_, consistent with the smaller radius of Lu^3+^. The dimeric structure results in a much smaller O–Lu–O angle of 86.9(1)° in **1**_**Lu**_. The intramolecular Lu···Fe separation is 5.0703(6) Å, and the Fe···Fe and Lu···Lu separations are 7.2193(12) Å and 7.1339(5) Å, respectively.

Despite the differences in the solid-state structures of **1**_**Gd**_ and **1**_**Lu**_, their FTIR spectra are similar, with both featuring two strong absorptions for the symmetric and antisymmetric stretches of the carbonyl ligands (Figure S2). The CO stretching vibrations occur at 1769 and 1694 cm^–1^ for solid **1**_**Gd**_ and at 1779 and 1728 cm^–1^ for **1**_**Lu**_, both of which are somewhat lower than the carbonyl bands of 1880 and 1735 cm^–1^ reported for K­[Fp] (in Nujol).^35^ These FTIR bands appear at similar energies to those found in other rare-earth isocarbonyl complexes, with the lower values of the stretching frequencies likely due to polarization of CO ligand via the Ln–O interactions.^36,37^ The ^1^H NMR spectrum of **1**_**Lu**_ in THF-D_8_ consists of sharp singlets at δ = 1.96 and 4.44 ppm for the for C_5_Me_5_ and C_5_H_5_ ligands (Figure S4), respectively, and the ^13^C­{^1^H} NMR spectrum shows resonances for the Cp* carbon atoms at 11.50 ppm (methyl) and 79.22 ppm (cyclopentadienyl) and for the Cp ligand at 119.12 ppm (Figure S5). The carbonyl ligands could not be detected by ^13^C NMR spectroscopy.

The reactions of **1**_**Gd**_, **1**_**Dy**_, and **1**_**Lu**_ with the readily reducible benchmark substrate phenazine^32^ (phnz, −0.364 V vs SCE) in benzene each produced red crystals of the dimetallic phenazine-bridged complexes [{(Cp*)_2_M}_2_(μ-phnz)] (**2**_**M**_) in yields of 21%, 56% and 33%, respectively (Scheme 1). Molecules of **1**_**M**_ are essentially isostructural (as reflected in their similar FTIR spectra, Figure S3), consisting of centrosymmetric dimers with two η^5^-Cp* ligands and a κ^1^-phnz ligand bound to each metal via the nitrogen atoms, with the heterocycle adopting a canted orientation (Figures 3 and S1, Tables S3 and S4). The Cp*–M–Cp* angles in **2**_**Gd**_, **2**_**Dy**_, and **2**_**Lu**_ are 137.27(1), 137.90(1), 137.87(3)°, respectively and, while there is minimal variation in the metallocene bending angle, the M–(Cp*)_cent_ distances decrease from 2.4060(2)/2.3844(2) Å in **2**_**Gd**_, to 2.3728(2)/2.3431(2) Å in **2**_**Dy**_ and 2.2920(9)/2.3108(1) Å in **2**_**Lu**_. Similarly, the M–N1 distances decrease in the order 2.340(3) Å, 2.310(4) Å and 2.245(16) Å in **2**_**Gd**_, **2**_**Dy**_, and **2**_**Lu**_, respectively, as expected based on the lanthanide contraction. The effects of reducing phenazine to the formally dianionic ligand [phnz]^2–^ are evidenced by the pattern of C–C bonds in the C_6_ rings. Using **1**_**Gd**_ as an example, the C–C distances are in the range 1.372(6)–1.427(5) Å (average 1.400 Å) and, from C21–C22, adopt a “short–long” alternation pattern, in contrast to the “long–short” pattern observed in phenazine itself.^38^ An increase in the C–N distances in **1**_**Gd**_ from 1.387(5)/1.394 Å by 0.046–0.052 Å relative to phenazine is also observed, indicating a change in the electronic structure consistent with population of ligand π* orbitals. The ^1^H and ^13^C­{^1^H} NMR spectra of **2**_**Lu**_ in toluene-D_8_ are consistent with the solid-state structure, with the former showing resonances at 5.96 and 4.53 ppm for the [phnz]^2–^ protons, and at 2.13 ppm for the Cp* methyl groups (Figure S6). The ^13^C­{^1^H} NMR spectrum of **2**_**Lu**_ features the expected five resonances, which occur at 10.7, 104.5, 118.7, 119.5, and 145.2 ppm (Figure S7).

**Figure 3:**
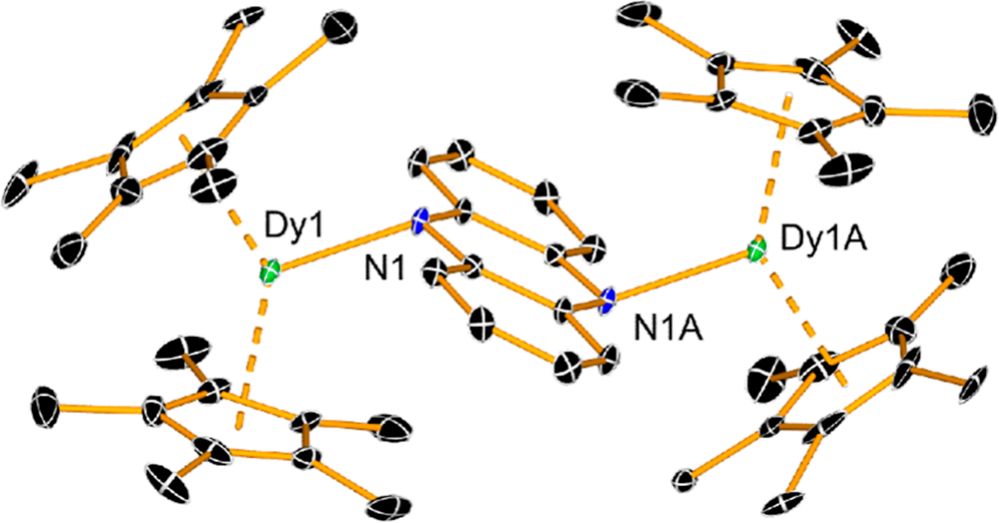
Molecular structure of **2**_**Dy**_. Hydrogen atoms are omitted for clarity, and thermal ellipsoids are set to 30% probability.

### Magnetic Properties

The molar magnetic susceptibility (χ_M_) properties of **1**_**Gd**_, **2**_**Gd**_ and **2**_**Dy**_ were determined in an applied DC field of 1000 Oe in the temperature range 2–300 K (Figure 4). For **1**_**Gd**_, χ_M_ *T* takes a value of 7.88 cm^3^ K mol^–1^ at 300 K and remains almost temperature independent down to 2 K, where a value of 7.44 cm^3^ K mol^–1^ is reached. The value of χ_M_ *T* for **2**_**Gd**_ at 300 K is 14.37 cm^3^ K mol^–1^, with a low-temperature downturn resulting in the susceptibility reaching 12.33 cm^3^ K mol^–1^ at 2 K. In both gadolinium compounds, the value of χ_M_ *T* at 300 K is close to the theoretical values of 7.88 cm^3^ K mol^–1^ and 15.76 cm^3^ K mol^–1^ expected for one and two Gd^3+^ ions (4f,^7^ ^8^S_7/2_ ground term), respectively,^39^ and the weak temperature dependence of the susceptibility is typical of very weak antiferromagnetic exchange interactions with neighboring ions. The field-dependence of the magnetization, *M*(*H*), for **1**_**Gd**_ at 1.9 K and 7 T is 6.92 μ_B_ (Figure S8), close to the expected saturation value of 7.0 μ_B_ for Gd^3+^, assuming *g* = 2.00 and S = 7/2. The *M*(*H*) plot for **2**_**Gd**_ reaches a magnetization value of 12.95 μ_B_ at 1.9 K and 7 T (Figure S8), slightly below the theoretical saturation value of 13.86 μB expected for two Gd^3+^ ions with *g* = 1.98 (see below), but within the normal range for gadolinium dimers.^40,41^

**Figure 4:**
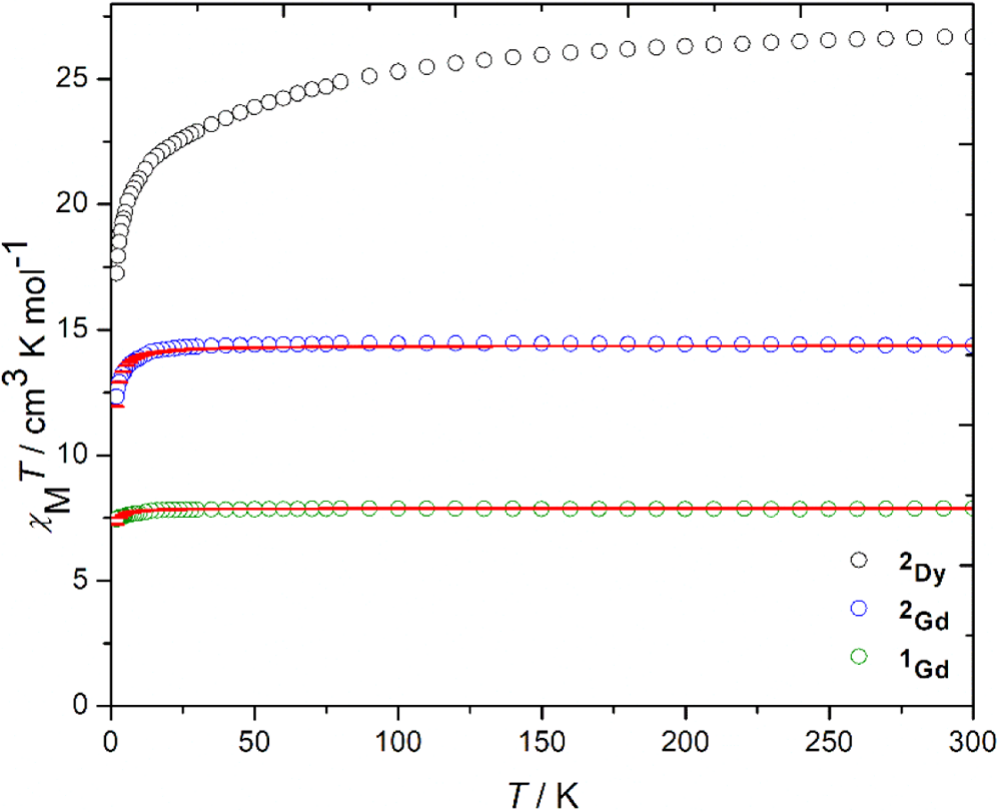
χ_M_ *T*(*T*) plots for **1**_**Gd**_ (green circles), **2**_**Gd**_ (black circles) and **2**_**Dy**_ (blue circles) in an applied field of 1000 Oe. Red lines represent fits to the data using eq 1 and the parameters stated in the text.

To gain further insight into the magnetic properties, fits of the susceptibility and magnetization data for both gadolinium compounds was obtained using the spin Hamiltonian in eq 1,^42^ where *J* is the exchange coupling constant, the ${Ŝ}_{Gd}$ terms denote the spin operators on each Gd^3+^ ion, β is the Bohr magneton, the *g*_Gd_ terms are the Landé *g*-values of each Gd^3+^ ion, and *B* is the magnetic field.

1 $$Ĥ=-2J\left({Ŝ}_{Gd1}\cdot {Ŝ}_{Gd2}\right)+\beta \left(g_{Gd1}\cdot {Ŝ}_{Gd1}+g_{Gd2}\cdot {Ŝ}_{Gd2}\right)\cdot B$$

A good fit of the susceptibility and magnetization data of **1**_**Gd**_ was obtained using *J* = – 0.009 cm^–1^ and *g* = 2.00, and for **2**_**Gd**_ a good fit was obtained using *J* = – 0.02 cm^–1^ and *g* = 1.98. Thus, in both compounds, the Gd^3+^ ions are coupled through extremely weak antiferromagnetic exchange, with the weaker coupling in **1**_**Gd**_ likely due to the greater distances between the metal centers.

The χ_M_ *T* profile for **2**_**Dy**_ shows a more pronounced temperature dependence. At 300 K, the value of χ_M_ *T* is 26.67 cm^3^ K mol^–1^, close to the expected value of 28.34 cm^3^ K mol^–1^ for two Dy^3+^ ions (4f,^9^ ^6^H_15/2_ ground term).^39^ Upon cooling to 75 K, the susceptibility decreases gradually before decreasing more sharply at lower temperatures and reaching 17.25 cm^3^ K mol^–1^ at 2 K. This behavior is consistent with progressive depopulation of the excited crystal field levels within the ^6^H_15/2_ multiplet and may also indicate magnetic blocking. The magnetization for **2**_**Dy**_ at 1.9 K shows a steep increase in fields up to about 2 T before increasing more slowly to reach a value of 9.80 μ_B_ (4.90 μ_B_ per Dy^3+^), consistent with saturation of the lowest Kramers doublet with *m*_J_ = ± 15/2 (theoretical value of 5.2 μ_B_) (Figure S10). Thus, AC susceptibility measurements were undertaken using a Physical Property Measurement System to check for SMM properties.

In zero applied DC field and an AC field of 5 Oe, the real (χ′) and imaginary (χ″) components of the AC susceptibility of **2**_**Dy**_ were measured as a function of frequency (ν). The plot of χ″(ν) displays maxima in the temperature range 2.5–10.5 K, with the position of the maximum remaining almost frequency independent up to 5 K (Figures 5 and S11). With increasing temperature, the maxima in χ″(ν) shift to higher frequencies before reaching the limit of the magnetometer at a temperature of 10.5 K. These data suggest that the magnetic moment of **2**_**Dy**_ relaxes via quantum tunnelling of the magnetization (QTM) at low temperatures and via an activated process (or processes) at higher temperatures. To understand the dynamic magnetism of **2**_**Dy**_, Cole–Cole plots of χ″(χ′) were constructed and used to extract magnetic relaxation times (τ) using a generalized Debye model and eqs S1 and S2 (see Supporting Information for full details). The Cole–Cole plots appear as depressed semicircles and were fitted using α-parameters in the range 0.11–0.22, indicating a relatively narrow distribution of relaxation times (Figure S12, Tables S5 and S6), the modest broadening being consistent with the coexistence of phonon-assisted and QTM pathways. Plotting ln (τ) as a function of *T* ^–1^ shows that the higher-temperature relaxation does not enter a well-defined linear regime (Figure 5), suggesting that Raman relaxation is dominant above 5 K. A fit of ln (τ) vs *T* ^–1^ was therefore achieved using eq 2, where *C* and *n* are the Raman coefficient and Raman exponent, respectively, and τ_QTM_^–1^ is the rate of QTM.

2 $$\tau^{-1}=CT^{n}+\tau_{QTM}^{-1}$$

**Figure 5:**
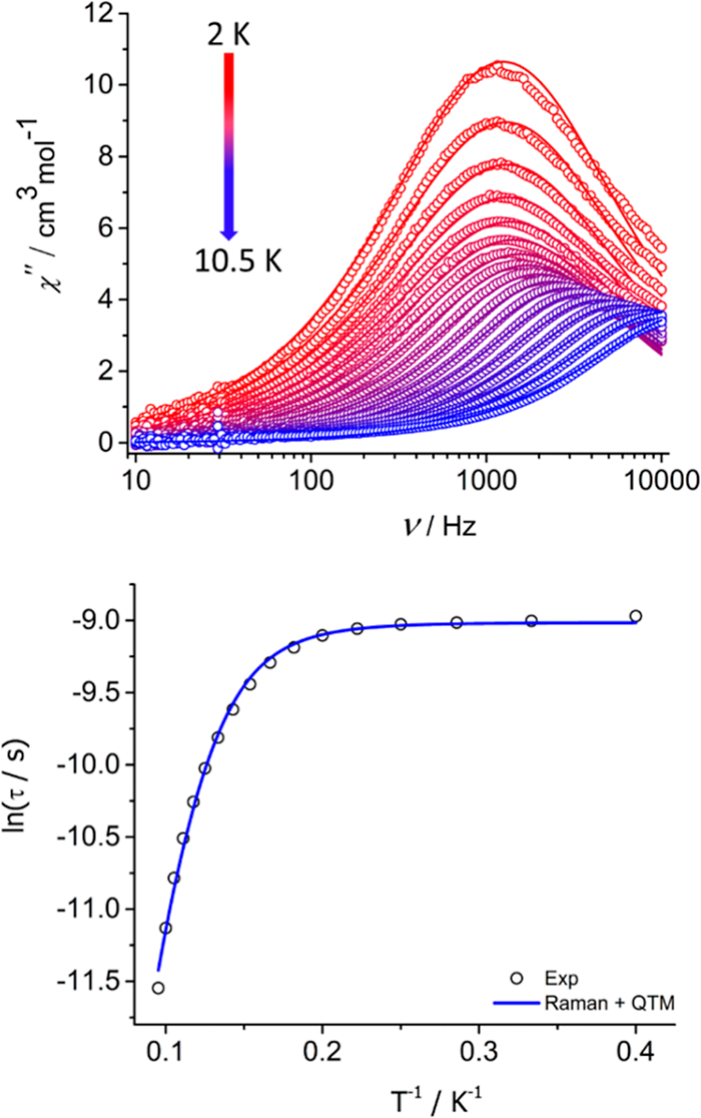
Upper: frequency dependence of the imaginary component of the AC susceptibility for **2**_**Dy**_ in zero DC field. Lower: relaxation times for **2**_**Dy**_ as a function of *T* ^–1^. Solid lines in both plots are fits to the data using the parameters stated in the text.

A good fit of the relaxation time data was achieved using *C* = 0.023 ± 0.011 s^–1^ K^–*n*^, *n* = 6.41 ± 0.21, and τ_QTM_ = 1.21 × 10^–4^ ± 1.93 × 10^–6^ s. The fitted Raman parameters are consistent with a conventional two-phonon Raman relaxation mechanism characteristic of a Kramers system, with the value of *C* indicating a moderate spin-phonon coupling strength and *n* reflecting relaxation in which the crystal field experienced by Dy^3+^ is modulated by acoustic phonons.^43,44^

The absence of an effective energy barrier (*U*_eff_) for **2**_**Dy**_, corresponding to an Orbach relaxation process, contrasts to the SMM properties observed for a variety of dysprosium metallocene complexes.^45–53^ However, this behavior can be explained by considering the Dy–Cp* distances in **2**_**Dy**_ relative to the Dy–N distances. Generally, a dominant axial crystal field originating from the cyclopentadienyl ligands is favored if the Dy–Cp* distances are shorter than the bond distances to the equatorial ligands, i.e., the phenazine nitrogen atoms in **2**_**Dy**_, facilitating Orbach relaxation. However, the molecular structure of **2**_**Dy**_ reveals that the Dy–N distances are shorter by approximately 0.03–0.06 Å, implying a dominant equatorial crystal field, in agreement with the absence of a measurable effective barrier. The dominant equatorial crystal field in **2**_**Dy**_ and the rapid QTM at low temperatures also impacts severely on the magnetic hysteresis properties of this complex, with extremely narrow *M*(*H*) loops being observed only at 3 K (Figure S13). The dynamic magnetic properties of **2**_**Dy**_ are thus broadly consistent with the closely related compound [{(Cp^ttt^)_2_Dy}_2_(μ-phnz)], which also does not display a measurable effective energy barrier to magnetization reversal, further highlighting the deleterious role of the equatorial phenazine dianion ligand.^33^

## Conclusions

In this study, we have expanded the chemistry of isocarbonyl-bridged rare-earth complexes as masked divalent reductants to include gadolinium and lutetium, metals for which authentic divalent compounds remain scarce. Salt metathesis reactions produced the isocarbonyl-bridged complexes **1**_**Gd**_ and **1**_**Lu**_, revealing that the structural motif adopted by complexes of the type {(Cp*)_2_M­(μ-Fp)} is sensitive to ionic radius, with the gadolinium version forming a coordination polymer and lutetium yielding a dimer. Despite these solid-state differences, spectroscopic data confirm comparable isocarbonyl bonding, consistent with substantial back-donation from iron into the CO ligands. All three complexes **1**_**M**_ (M = Gd, Dy, Lu) are effective two-electron reductants toward phenazine, delivering the dimetallic phenazine-bridged complexes **2**_**M**_ and demonstrating that masked divalent reactivity can be extended across a wide range of lanthanide ionic radii. Structural metrics and NMR data are consistent with reduction of phenazine to its dianionic form, stabilized by strong M–N interactions, underscoring the ability of *N*-heterocyclic ligands to serve as electron reservoirs in lanthanide chemistry.

Magnetic measurements show that **1**_**Gd**_ and **2**_**Gd**_ exhibit extremely weak antiferromagnetic exchange, reflecting the long metal–metal separations. The dysprosium–phenazine complex **2**_**Dy**_ displays slow magnetic relaxation dominated by Raman and quantum tunnelling processes, but without a measurable Orbach barrier due to a dominant equatorial crystal field imposed by the phenazine ligand. These results illustrate how redox-active heterocycles can influence magnetic relaxation pathways beyond those commonly found in dysprosium metallocene SMMs.

## Supplementary Material



## Data Availability

Other data that support the findings of this study are openly available at DOI: 10.25377/sussex.31239454.

## Notes

General considerations and synthesis details; X-ray crystallography; IR spectra; NMR spectra; Magnetic property measurements (PDF)

## ∥

Inorganic and Organometallic Chemistry, Universität Erlangen-Nürnberg, Egerlandstrasse 1, 91058 Erlangen, Germany
