# Supplementary material for: Expanding the Reactivity of Masked Divalent Lanthanide–Isocarbonyl Complexes
Source: Organometallics. 2026 Mar 31;45(7):885–91. doi: 10.1021/acs.organomet.6c00042 (PMC13081112; doi:10.1021/acs.organomet.6c00042)
Supplement: Supplementary file 1 [file om6c00042_si_001.pdf]

Expanding the Reactivity of Masked Divalent Lanthanide-Isocarbonyl Complexes

Siobhan R. Temple,[a] Arpan Mondal,[a] Sean R. Giblin,[b] Jinkui Tang,[c] and Richard A. Layfield*[a]

a Department of Chemistry, School of Life Sciences, University of Sussex, Brighton, BN1 9RH, UK. Email: r.layfield@sussex.ac.uk
b School of Physics and Astronomy, Cardiff University, Cardiff CF24 3AA, U.K.
c Changchun Institute of Applied Chemistry, Chinese Academy of Sciences, Renmin Street 5626, 130022 Changchun, China.

Contents

General Considerations	S1
X-ray crystallography	S3
IR spectra	S7
NMR spectra	S9
Magnetic Property Measurements	Ss111
References	S14

General considerations.
All manipulations were performed under anaerobic and anhydrous conditions using Schlenk and glovebox techniques. Solvents were refluxed over a suitable drying agent (molten potassium for THF, benzene, toluene, toluene-D_8, THF-D_8 and Na/K alloy for hexane) for a minimum of three days before use. Solvents were distilled, degassed (minimum of three freeze-pump-thaw cycles), and stored in ampoules over potassium mirrors (benzene, toluene, hexane) or activated 4 Å molecular sieves (THF). NMR spectra were recorded on a Varian VNMR S400 spectrometer at 303 K unless otherwise stated (frequencies 400 MHz for ^{1}H and 100 MHz for ^{13}C). FTIR spectra were recorded on a Bruker Alpha spectrometer with platinum-diamond ATR module. Elemental analyses were carried out at Elemental Microanalysis (Okehampton, UK) or Mikroanalytiches Labor Pascher (Remagen, Germany). Literature procedures were used to prepare [Cp*$_2$M][BPh$_4$] (M = Gd, Dy, Lu),[1] [{(η^5-Cp*)$_2$Dy}(μ-CO)$_2$Fe(η^5-Cp)]$_2$ and KFp.[2]

Synthesis of [(Cp*)$_2$Gd(THF){(μ-OC)FeCp(μ-CO)}]$_\infty$ (1_{Gd})
A solution of KFp (90.0 mg, 0.42 mmol) in THF (5 mL) was added to a solution of [Cp*$_2$Gd(BPh$_4$)] (311.0 mg, 0.42 mmol) in THF (5 mL) at room temperature. Upon addition, the mixture developed an orange colour and a precipitate formed. The reaction was filtered, layered with hexane and, after storage at room temperature overnight, orange crystals of **1**$_{Gd}$ formed (123.0 mg, 43 %). Elemental analysis (%): Found (calculated) for $C_{58}H_{78}Fe_2Gd_2O_5$:C 54.13 (54.36), H 6.30 (6.13). FTIR ($\tilde{\nu}$/cm^1): 3015, 2980, 2902, 2863, 1796 (CO stretch), 1694 (CO stretch), 1432, 1009, 860, 796, 682, 602, 579, 498.

Synthesis of [(Cp*)$_2$Lu{(μ-OC)FeCp(μ-CO)}]$_2$ (1_{Lu})
Compound **1**$_{Lu}$ was synthesised using the procedure described for **1**$_{Gd}$, with [Cp*$_2$Lu(BPh$_4$)] (599.0 mg, 0.78 mmol) and KFp (169.0 mg, 0.78 mmol) which yielded orange crystals of **1**$_{Lu}$ (243.0 mg, 50 %). FTIR ($\tilde{\nu}$/cm^1): 3073, 2974, 2917, 2846, 1796 (CO stretch), 1728 (CO stretch), 1427, 1250, 1094, 1023, 810, 711, 569, 512. ^{1}H NMR (400 MHz, THF-D_8, δ/ppm): 4.44 (10H, s, Cp), 1.97 (60H, s, Cp*). ^{13}C{^{1}H} NMR (100 MHz, THF-D_8, δ/ppm): 119.12 (Cp* $\underline{C}$CH$_3$), 79.20 (Cp), 11.50 (Cp* C$\underline{C}$H$_3$). Found (calculated) for $C_{54}H_{70}Fe_2Lu_2O_4$: C 50.56 (52.10), H 5.20 (5.67). Despite multiple attempts, carbon analysis to within 0.4% could not be obtained.

Synthesis of [{(Cp*)$_2$Gd}$_2$(μ-phnz)] (2$_{Gd}$)

Phenazine (16.4 mg, 0.090 mmol) was added to a suspension of **1$_{Gd}$** (123.0 mg, 0.18 mmol) in benzene (2 mL) at room temperature. The solution was swirled and left to stand at room temperature for three days, which yielded red crystalline material. The solution was decanted and washed with (3 × 5 mL) hexane before drying under reduced pressure to give red crystals of **2$_{Gd}$** (21.4 mg, 21 %). FTIR ($\tilde{\nu}$/cm^{1}): 3035, 2971, 2908, 2837, 1582, 1448, 1373, 1325, 1275, 1205, 1106, 1018, 893, 795, 727, 670, 587. Elemental analysis (%): Found (calculated) for $C_{52}H_{68}N_2Gd_2$: C 61.30 (60.31), H 6.02 (6.62), N 2.58 (2.71). Despite multiple attempts, carbon analysis to within 0.4% could not be obtained.

Synthesis of [{(Cp*)$_2$Dy}$_2$(μ-phnz)] (2$_{Dy}$)

Compound **2$_{Dy}$** was synthesised using the procedure described for **2$_{Gd}$**, using **1$_{Dy}$** (198.0 mg, 0.16 mmol) and phenazine (29.0 mg, 0.16 mmol). Compound **2$_{Dy}$** was isolated red crystals (101.0 mg, 56 %). Elemental analysis (%): Found (calculated) for $C_{52}H_{68}N_2Dy_2$: C 58.87 (59.70), H 6.39 (6.55), N 2.39 (2.68). FTIR ($\tilde{\nu}$/cm^{1}): 3051, 2967, 2903, 2853, 1591, 1462, 1385, 1335, 1278, 1215, 1123, 1023, 896, 796, 718,669, 598. Despite multiple attempts, carbon analysis to within 0.4% could not be obtained.

Synthesis of [{(Cp*)$_2$Lu}$_2$(μ-phnz)] (2$_{Lu}$)

Compound **2$_{Lu}$** was synthesised using the procedure described for **2$_{Gd}$**, using **1$_{Lu}$** (40.8 mg, 0.033 mmol) and phenazine (6.0 mg, 0.033 mmol). Compound **2$_{Lu}$** was isolated as red crystals (12.6 mg, 33 %). FTIR ($\tilde{\nu}$/cm^{1}): 3055, 2971, 2899, 2853, 1591, 1456, 1329, 1282, 1215, 1124, 1019, 894, 708. ^{1}H NMR (toluene-D$_8$, δ/ppm): 5.96 (m, 4H, CH-phen), 4.52 (m, 4H, CH-phen), 2.13 (s, 60 H, Cp*). ^{13}C{^{1}H} NMR (toluene-D$_8$, δ/ppm): 145.16 (phenazine, N<u>C</u>CH); 119.47(Cp* <u>C</u>CH$_3$), 118.66 (phenazine, <u>C</u>HCH), 104.53 (phenazine, <u>C</u>HCH), 10.65 (Cp* C<u>C</u>H$_3$). Despite numerous attempts, satisfactory elemental analysis could not be obtained for $C_{52}H_{68}N_2Lu_2$. Evidence for the bulk purity of **2$_{Lu}$** was obtained from the NMR spectra (Figures S6-S7) and similarities in the FTIR spectra (Figure S3) of **2$_{Gd}$**, **2$_{Dy}$** and **2$_{Lu}$**.

X-Ray crystallography

Data were collected on a Rigaku FR-007HF rotating anode diffractometer equipped with Saturn 724+ CCD area detector or an Agilent Gemini Ultra diffractometer using CuK$_\alpha$ radiation (λ = 1.54184 Å) and a quarter-chi goniometer performing ω scans to fill the Ewald sphere at 100 K. All structures were solved in Olex2 with SHELXL using intrinsic phasing and refined with least squares minimisation. Isotropic parameters were used for the hydrogen atoms and anisotropic thermal parameters were used for non-hydrogen atoms.[3–5]

Table S1. Crystal data and structure refinement for **1$_{M}$**.

	**1$_{Gd}$**	**1$_{Lu}$**
CSD reference	2479152	2479153
Empirical formula	$C_{62}H_{86}Fe_2Gd_2O_6$	$C_{54}H_{70}Fe_2Lu_2O_4$
Formula weight	1353.50	1244.74
T/K	100.00(10)	100.01(10)
Crystal system	Triclinic	Triclinic
Space group	$P\bar{1}$	$P\bar{1}$
a/Å	10.0709(2)	9.5696(2)
b/Å	16.5987(3)	10.0077(3)
c/Å	18.3984(4)	13.3077(2)
α/°	76.042(2)	96.136(2)
β/°	74.232(2)	98.696(2)
γ/°	79.217(2)	96.823(2)
V/Å^3	2847.99(11)	1240.74(5)
Z	2	2
ρ_{calc} / g cm^{-3}	1.578	1.666
F(000)	1372.0	620.0
Reflections collected	49869	20037
Independent reflections	10045	4374
Data/restraints/parameters	10045/456/669	4374/60/290
R_{int} / %	0.0916	0.0625
Goodness-of-fit on F^2	1.159	1.075
R_1[a]	0.0544	0.0340
wR_2[b]	0.1414	0.0873

[a]$R_1[I>2\sigma(I)] = \sum||F_o|-|F_c||/\sum|F_o|$; [b]$wR_2$ [all data] = $[\sum\{w(F_o^2 - F_c^2)\ 2\}/\sum\{w(F_o^2)^2\}]^{1/2}$

Table S2. Selected distances (Å) and angles (°) for the isocarbonyl-bridged complexes $\mathbf{1_M}$.

	$\mathbf{1_{Gd}}$	$\mathbf{1_{Dy}}$[a]	$\mathbf{1_{Lu}}$
M1−Cp*$_{cent}$	2.434(3)<br>2.428(3)	2.344(7)<br>2.339(7)	2.2816(18)<br>2.2822(19)
M2−Cp*$_{cent}$	2.4449(4)<br>2.4308(4)	-	-
M1−OC	2.410(4)<br>2.479(4)	2.293(9)<br>2.287(12)	2.220(3)<br>2.228(3)
M2−OC	2.417(4)<br>2.488(4)	-	-
M1−O(THF)	2.428(4)	-	-
M2−O(THF)	2.426(4)	-	-
Fe1−Cp$_{cent}$	1.748(3)	1.728(10)	1.733(3)
Fe2-Cp$_{cent}$	1.753(3)		
Fe1−CO	1.697(6)<br>1.710(6)	1.663(12)<br>1.681(18)	1.678(4)<br>1.686(4)
Fe2−CO	1.698(6)<br>1.720(7)	-	-
Cp*$_{cent}$−M1−Cp*$_{cent}$	136.78(9)	141.5(2)	141.70(6)
Cp*$_{cent}$−M2−Cp*$_{cent}$	138.333(13)		
CO−M1−OC	147.313)	87.5(4)	86.92(14)
CO−M2−OC	147.76(13)	-	-
OC−Fe1−CO	93.5(3)	88.8(7)	87.9(2)
OC−Fe2−CO	91.7(3)		

[a] Data from reference 6.

Table S3. Crystal data and structure refinement for $\mathbf{2_M}$.

	$\mathbf{2_{Gd}}$	$\mathbf{2_{Dy}}$	$\mathbf{2_{Lu}}$
CSD reference	2479154	2479155	2479156
Empirical formula	$C_{58}H_{74}N_2Gd_2$	$C_{58}H_{74}N_2Dy_2$	$C_{58}H_{74}N_2Lu_2$
Formula weight	1113.69	1124.19	1149.13
T/K	100.01(12)	100.00(11)	100.01(10)
Crystal system	triclinic	triclinic	triclinic
Space group	$P\bar{1}$	$P\bar{1}$	$P\bar{1}$
a/Å	10.5137(3)	10.5308(3)	10.5423(3)
b/Å	11.0034(2)	10.9448(2)	10.8484(2)
c/Å	11.2630(3)	11.2861(3)	11.3125(2)
α/°	79.760(2)	79.477(2)	79.064(2)
β/°	75.609(2)	75.521(3)	75.891(2)
γ/°	85.365(2)	85.092(2)	85.428(2)
V/Å^3	1241.07(6)	1237.22(6)	1231.20(5)
Z	1	1	1
ρ_{calc} / g cm^{-3}	1.490	1.509	1.550
F(000)	564.0	568.0	578.0
Reflections collected	41474	42432	20157
Independent reflections	4375	4353	4310
Data/restraints/parameters	4375/88/367	4353/126/356	4310/0/290
R_{int} / %	0.0408	0.0958	0.0223
Goodness-of-fit on F^2	1.034	1.035	1.115
R_1[a]	0.0315	0.0458	0.0174
wR_2[b]	0.0770	0.1176	0.0454

[a]$R_1[I>2\sigma(I)] = \sum||F_o|-|F_c||/\sum|F_o|$; [b]$wR_2$ [all data] = $[\sum\{w(F_o^2 - F_c^2)\ 2\}/\sum\{w(F_o^2)^2\}]^{1/2}$

Table S4. Selected bond lengths (Å) and angles (°) for the phenazine-bridged complexes $\mathbf{2_M}$.

	$\mathbf{2_{Gd}}$	$\mathbf{2_{Dy}}$	$\mathbf{2_{Lu}}$
M–Cp*$_{cent}$	2.4060(2)	2.3728(2)	2.2920(9)
M–Cp*$_{cent}$	2.3844(2)	2.3431(2)	2.3108(8)
M–N1	2.340(3)	2.310(4)	2.245(15)
C21–C22	1.372(6)	1.364(8)	1.367(3)
C22–C23	1.412(5)	1.413(7)	1.409(3)
C23–C24	1.398(5)	1.393(7)	1.387(3)
C24–C25	1.427(4)	1.436(6)	1.427(3)
C25–C26	1.386(5)	1.391(7)	1.404(3)
C26–C21	1.403(6)	1.400(8)	1.408(4)
N1–C24	1.387(5)	1.384(6)	1.389(3)
N1–C25	1.394(5)	1.388(6)	1.396(3)
Cp*$_{cent}$–M–Cp*$_{cent}$	137.27(7)	137.90(1)	137.87(3)

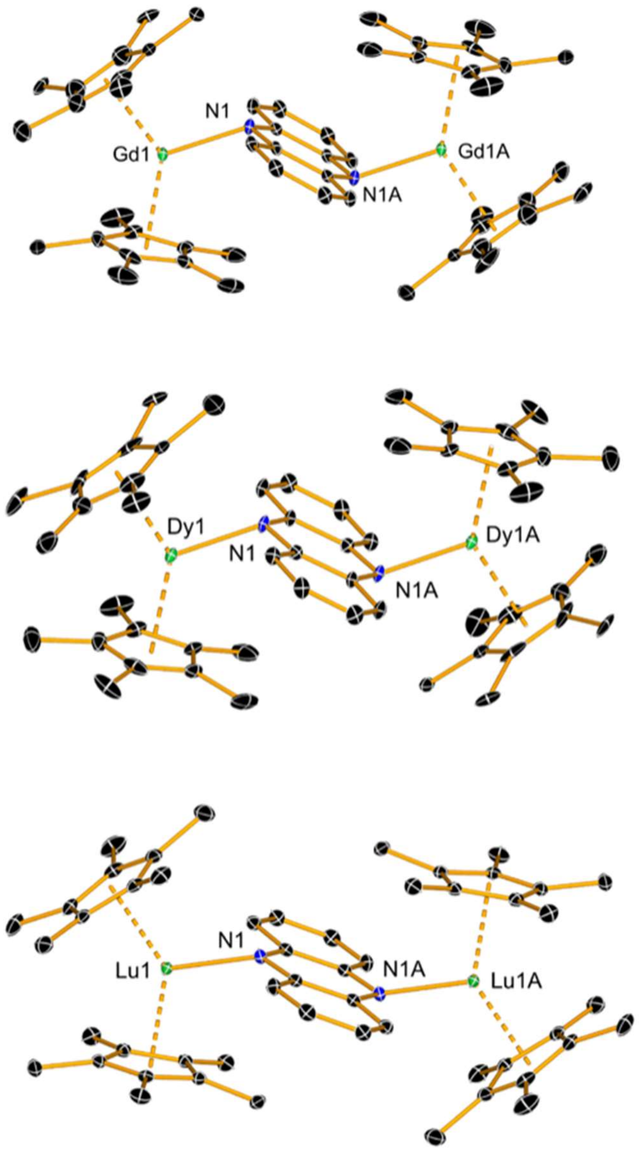

Figure S1. Molecular structures of $\mathbf{2}_{\mathbf{Gd}}$, $\mathbf{2}_{\mathbf{Dy}}$ and $\mathbf{2}_{\mathbf{Lu}}$. Thermal ellipsoid set at 30 % probability. Unlabelled black atoms are carbon. Hydrogen atoms are omitted for clarity.

FTIR Spectra

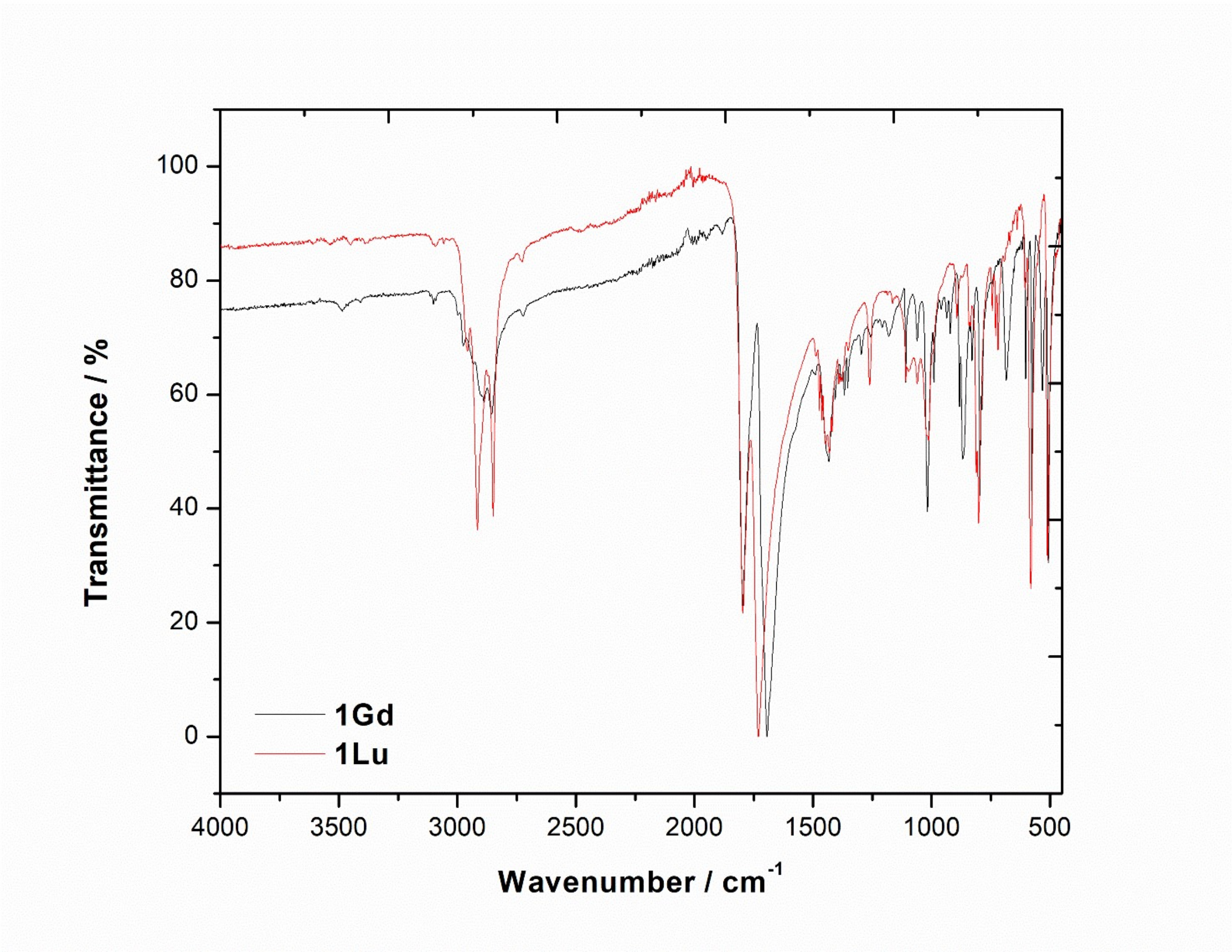

Figure S2. FTIR spectra of $\mathbf{1}_{Gd}$ (black) and $\mathbf{1}_{Lu}$ (red).

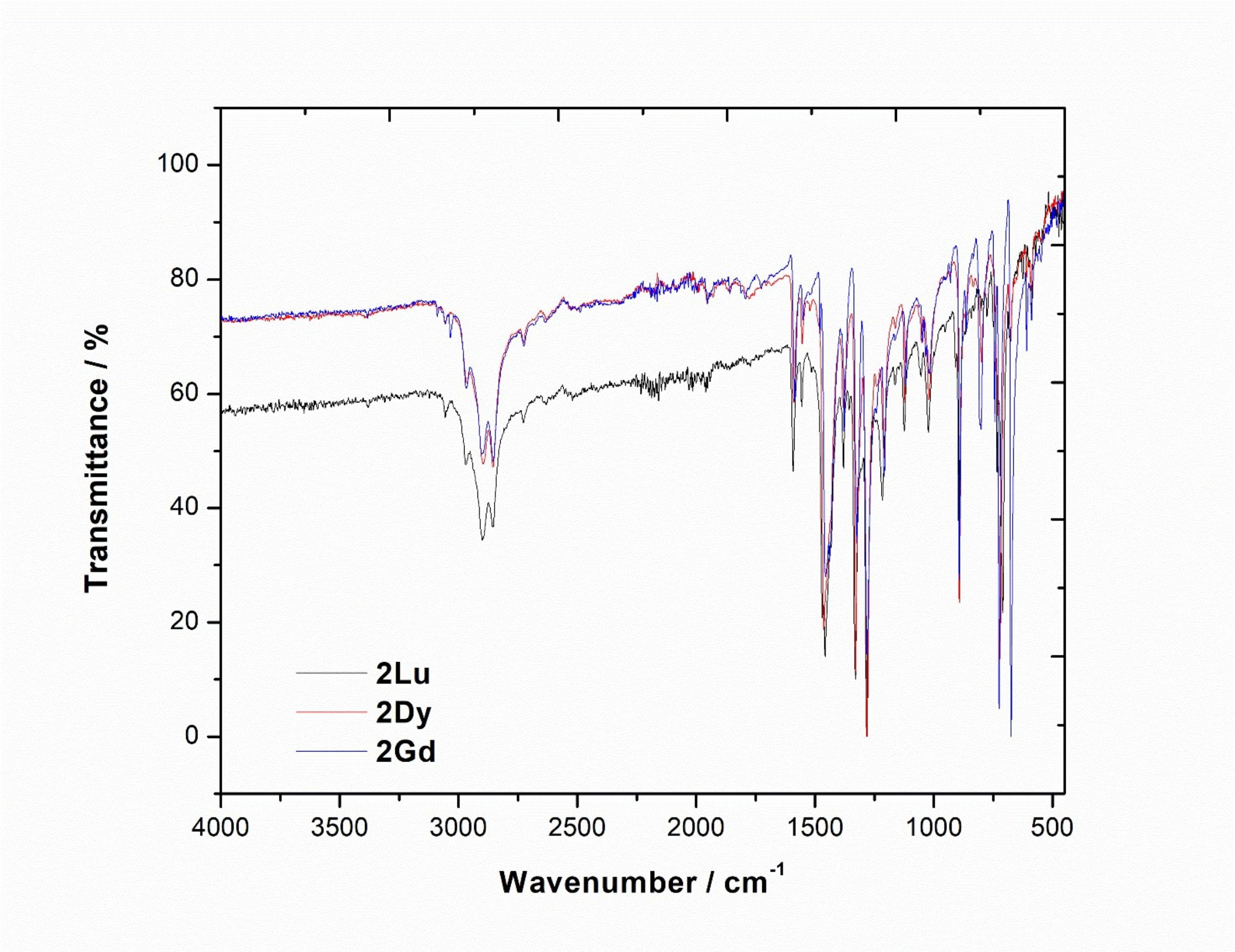

Figure S3. FTIR spectra of **2**$_{Gd}$ (blue), **2**$_{Dy}$ (red) and **2**$_{Lu}$ (black).

NMR Spectra

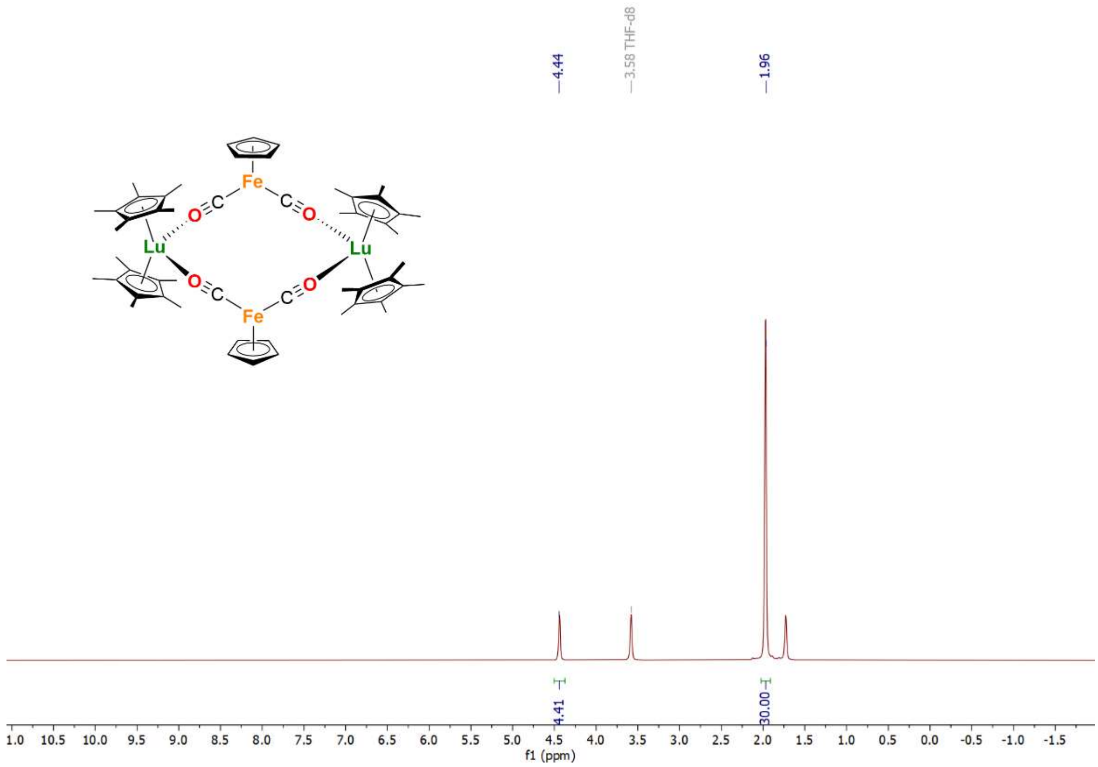

Figure S4. ^{1}H NMR spectrum of $\mathbf{1_{Lu}}$ in THF-D_8 at 303 K.

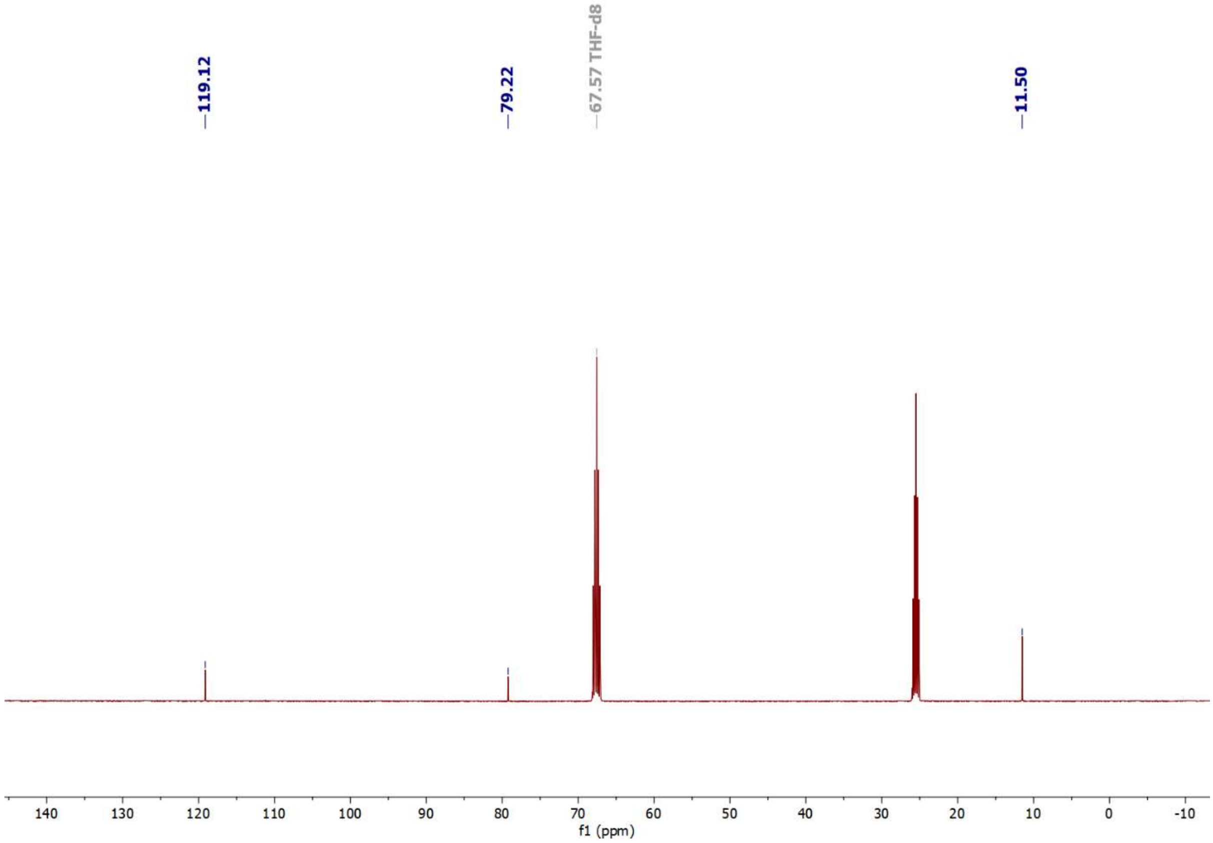

Figure S5. $^{13}C\{^{1}H\}$ NMR spectrum of $\mathbf{1_{Lu}}$ in THF-D_8 at 303 K.

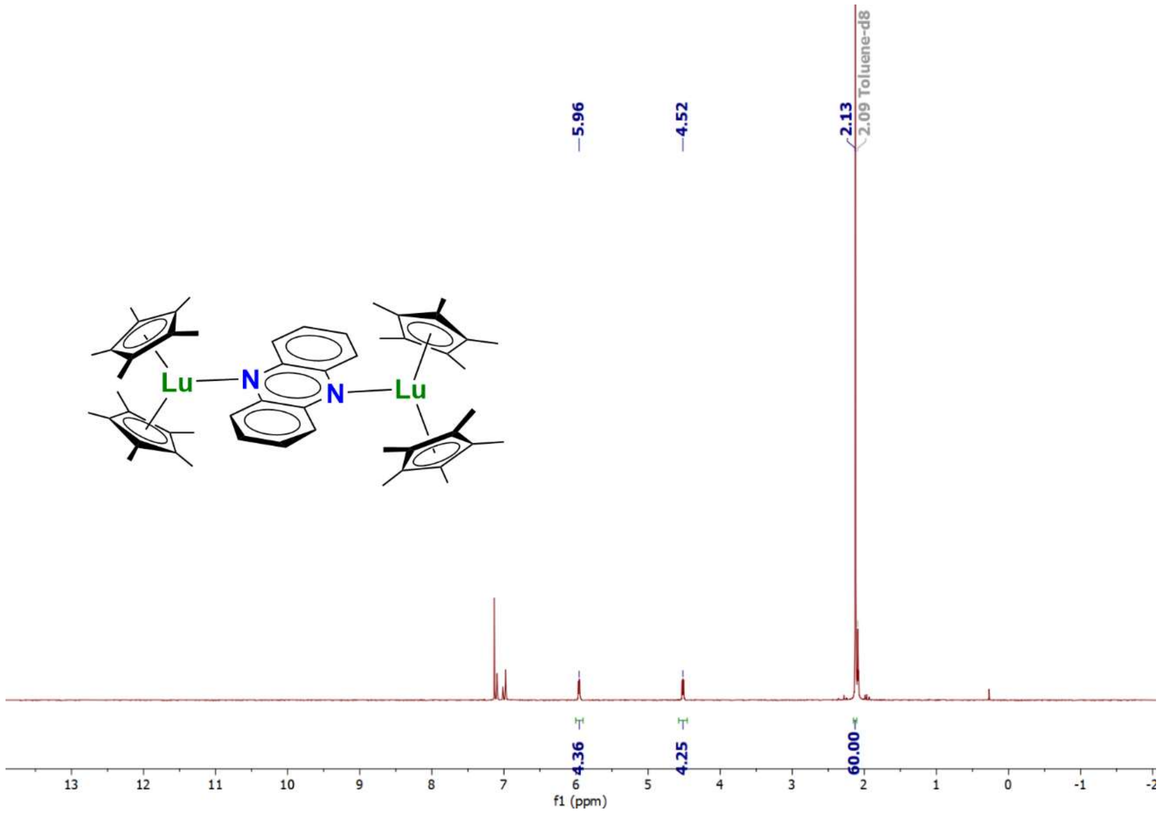

Figure S6. ^{1}H NMR spectrum of $\mathbf{2_{Lu}}$ in toluene-D$_8$ at 303 K.

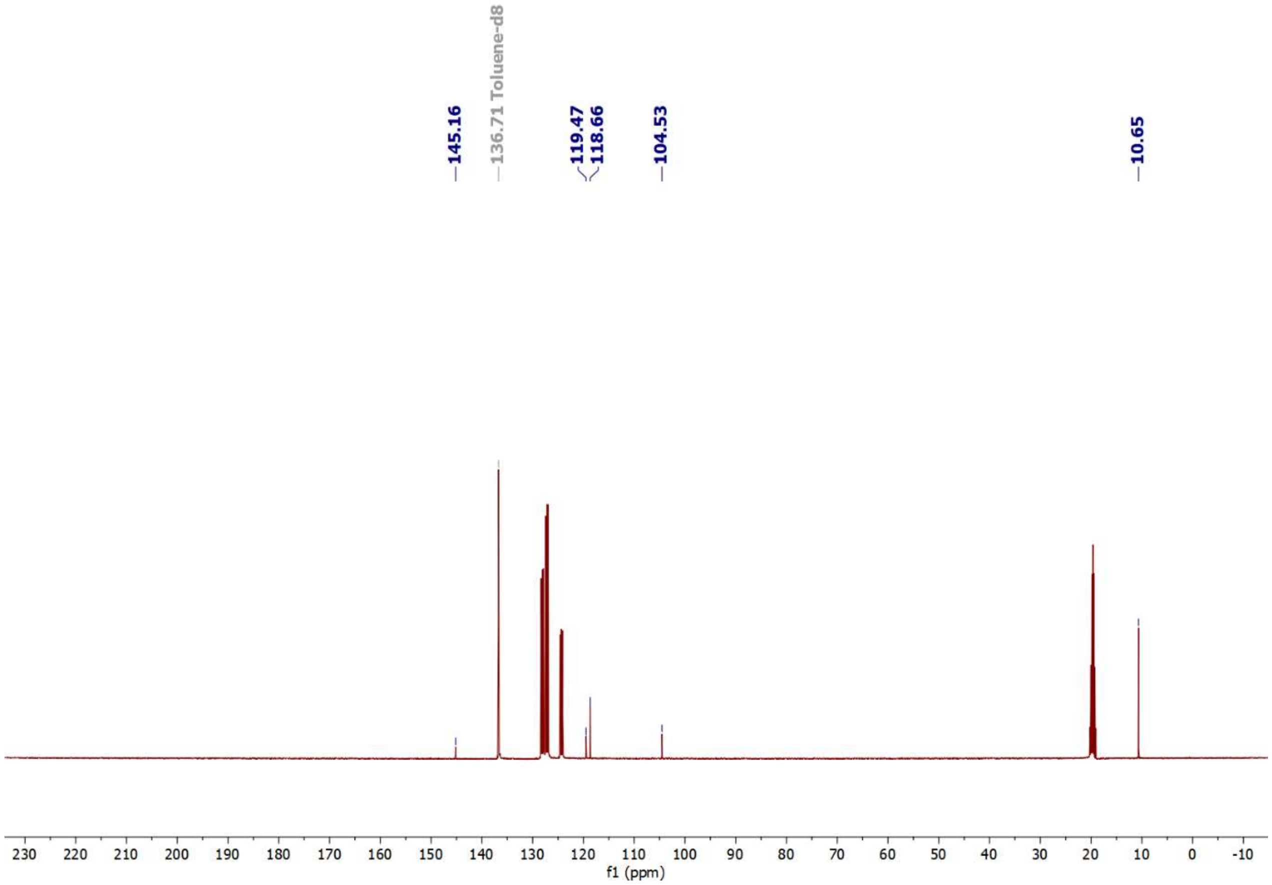

Figure S7. $^{13}C\{^{1}H\}$ NMR spectrum of $\mathbf{2_{Lu}}$ in toluene-D$_8$ at 303 K.

Magnetic Property Measurements

The DC magnetic properties of polycrystalline samples of **1**$_{Gd}$, **2**$_{Gd}$ and **2**$_{Dy}$ were measured using a QD MPMS-XL7 SQUID magnetometer equipped with a 7 T magnet. AC susceptibility measurements on **2**$_{Dy}$ were performed on a QD PPMS. Samples were restrained in eicosane and sealed in 7 mm NMR tubes. Direct current (DC) magnetic susceptibility measurements were performed in an applied field of 1000 Oe in the temperature range 1.9-300 K. AC susceptibility measurements were carried out in zero DC field with a 5 Oe oscillating field. Diamagnetic corrections were calculated using Pascals constants.[7]

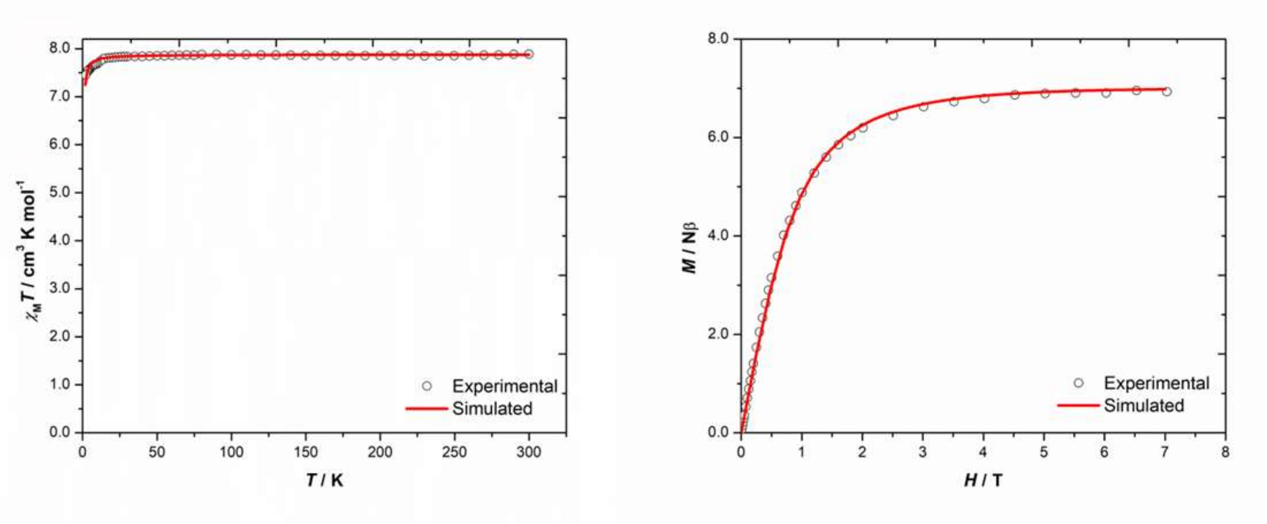

Figure S8. Plot of $\chi_M T(T)$ for **1**$_{Gd}$ in an applied field of 1000 Oe (left). Plot of field (H) dependence of the magnetization (M) at 1.9 K (right). The $\chi_M T(T)$ values at 300 K and 2 K are 7.88 cm^3 K mol^{-1} and 7.44 cm^3 K mol^{-1}, respectively. Red lines are fits of the data using the spin Hamiltonian in equation 1 in the main text.

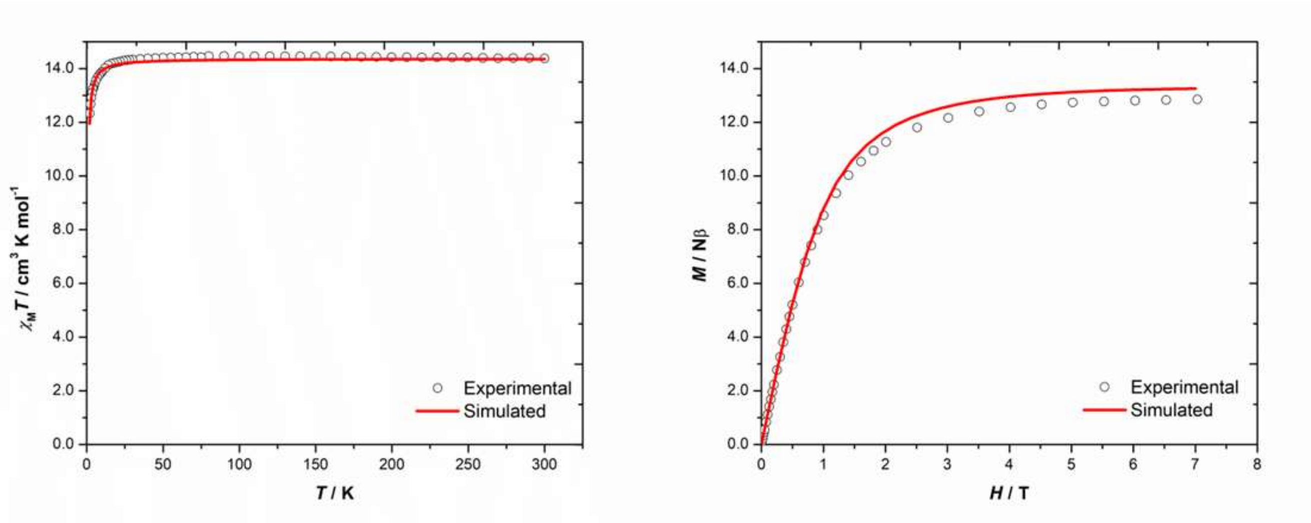

Figure S9. Plot of $\chi_M T(T)$ for **2**$_{Gd}$ in an applied field of 1000 Oe (left) and Field (M) dependence of the magnetization (M) at 2 K (right). The $\chi_M T$ values are 14.37 cm^3 K mol^{-1} at 300 K and 12.33 cm^3 K mol^{-1} at 2 K. Red lines are fits of the data using the spin Hamiltonian in equation 1 in the main text.

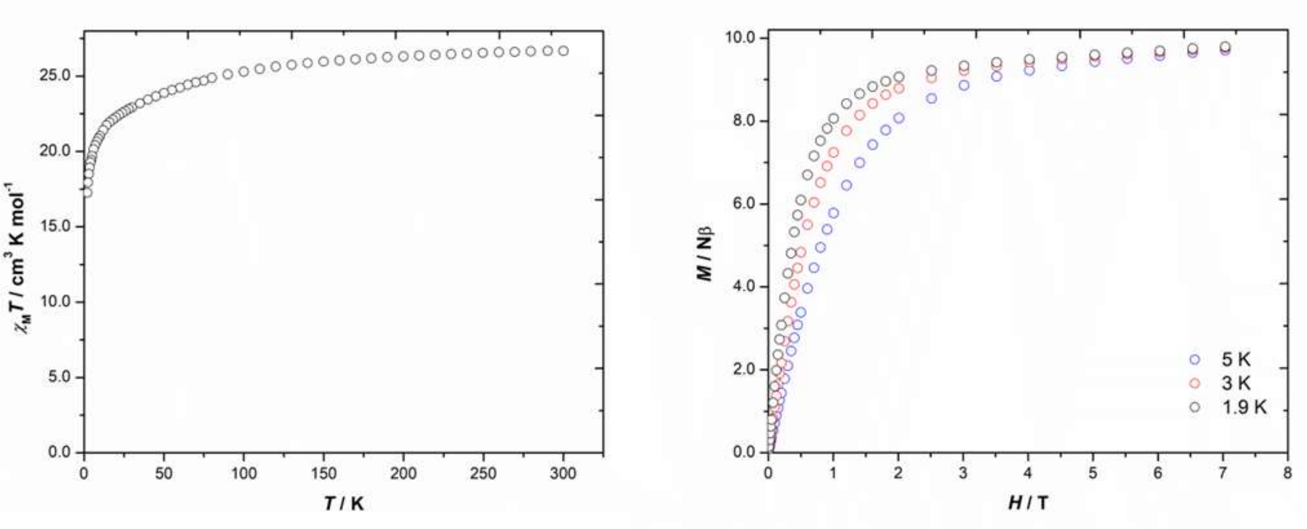

Figure S10. Plot of $\chi_M T(T)$ for $\mathbf{2_{Dy}}$ in an applied field of 1000 Oe (left) and field (*M*) dependence of the magnetization (*M*) at 1.9 K, 3 K and 5 K (right). The $\chi_M T$ values are 26.67 cm^3 K mol^{-1} at 300 K and 17.25 cm^3 K mol^{-1} at 2 K.

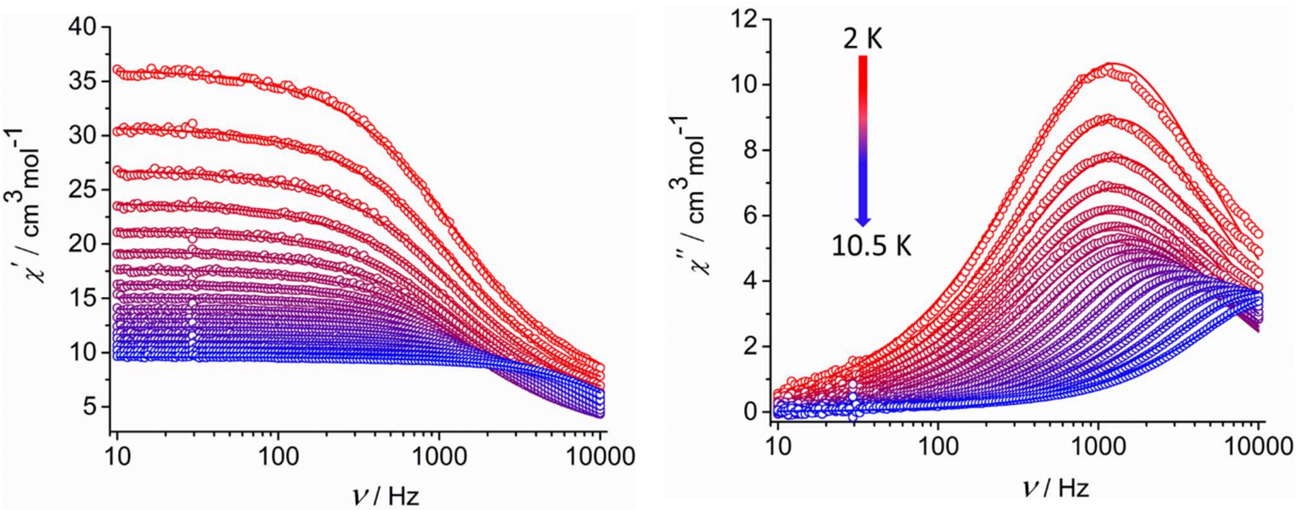

Figure S11. Left: frequency dependence of the real component of the AC susceptibility for $\mathbf{2_{Dy}}$. Right: frequency dependence of the imaginary component of the AC susceptibility for $\mathbf{2_{Dy}}$ in zero DC field. Measurements were conducted in zero DC field using an AC field of 5 Oe. Solid lines are fits to the data according to equations S1 and S2 and the parameters in Table S5.

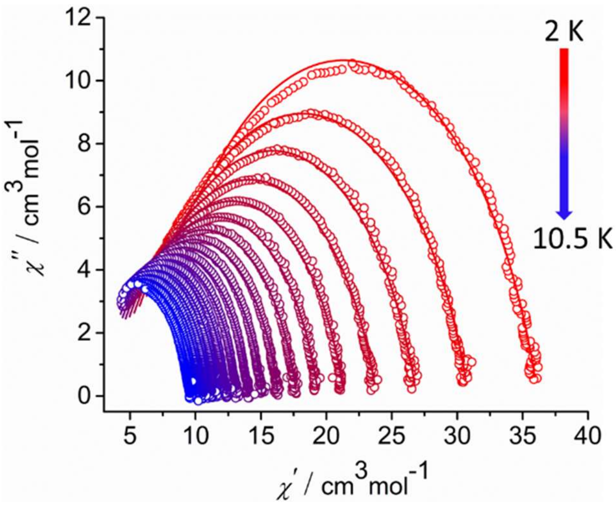

Figure S12. Cole-Cole plot of χ' (χ'') for $\mathbf{2_{Dy}}$. Solid lines are fits to the data according to equations S1 and S2 and the parameters in Table S5.

$$\chi'(\nu) = \chi_S + \frac{(\chi_T - \chi_S)[1 + (2\Pi\nu\tau)^{(1-\alpha)} Sin(\frac{\alpha\Pi}{2})]}{1 + 2(2\Pi\nu\tau)^{(1-\alpha)} Sin\left(\frac{\alpha\Pi}{2}\right) + (2\Pi\nu\tau)^{2(1-\alpha)}} \qquad \text{(Equation S1)}$$

$$\chi''(\nu) = \frac{(\chi_T - \chi_S)(2\Pi\nu\tau)^{(1-\alpha)} Cos(\frac{\alpha\Pi}{2})}{1 + 2(2\Pi\nu\tau)^{(1-\alpha)} Sin\left(\frac{\alpha\Pi}{2}\right) + (2\Pi\nu\tau)^{2(1-\alpha)}} \qquad \text{(Equation S2)}$$

Table S5. Relaxation fitting parameters for $\mathbf{2_{Dy}}$ corresponding to Figure S12.

***T* / K**	**χ_S / cm^3 mol^{-1}**	**χ_T / cm^3 mol^{-1}**	τ / s	α
2.5	6.4502	36.15207	1.27247E-4	0.2081
3	5.47898	30.85532	1.23089E-4	0.21757
3.5	4.91891	26.88199	1.21698E-4	0.21561
4	4.45188	23.81888	1.20293E-4	0.21541
4.5	4.14692	21.35138	1.16665E-4	0.20704
5	3.90466	19.38284	1.11281E-4	0.19482
5.5	3.64257	17.72667	1.02431E-4	0.18243
6	3.4185	16.31678	9.22299E-5	0.1656
6.5	3.19323	15.08531	7.94207E-5	0.14706
7	2.96736	14.03802	6.67135E-5	0.13062
7.5	2.73205	13.13702	5.48371E-5	0.118
8	2.47572	12.36433	4.43132E-5	0.1114
8.5	2.19413	11.67086	3.51326E-5	0.1094
9	1.86511	11.06552	2.7283E-5	0.11454
9.5	1.51499	10.50429	2.07234E-5	0.12079
10	0.87404	10.02323	1.46464E-5	0.14331
10.5	0.01023	9.57652	9.67199E-6	0.1686

Table S6. Relaxation time fitting parameters for $\mathbf{2_{Dy}}$.

Fitting equation	Parameters	
$\tau^{-1} = CT^n + \tau_{QTM}^{-1}$	τ_{QTM}	$1.21 \times 10^{-4} \pm 1.93 \times 10^{-6}$ s
	C	0.023 ± 0.011 s^{-1} K^{-n}
	n	6.41 ± 0.21

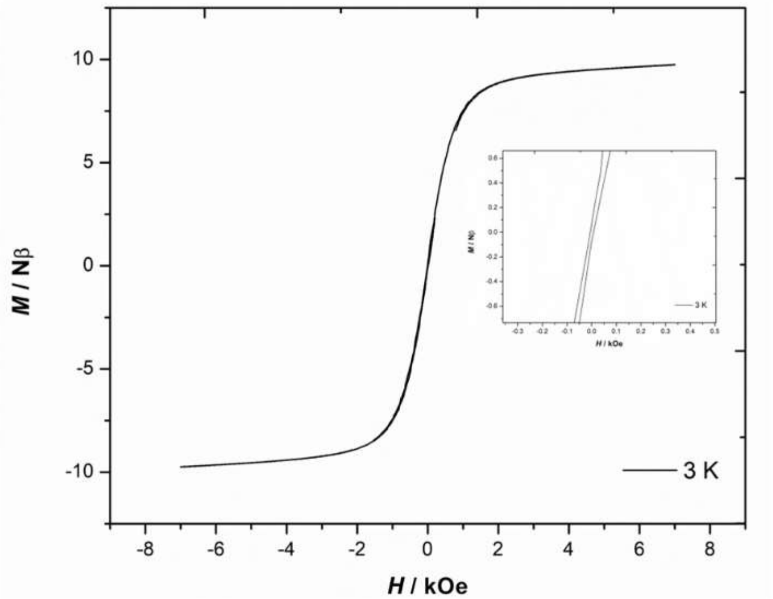

Figure S13. Magnetic hysteresis loop for $\mathbf{2_{Dy}}$ at 3 K.

References

1. Evans, W. J.; Seibel, C. A.; Ziller, J. W. Unsolvated Lanthanide Metallocene Cations [$(C_5Me_5)_2Ln$][BPh_4]: Multiple Syntheses, Structural Characterization, and Reactivity Including the Formation of $(C_5Me_5)_3Nd$. *J. Am. Chem. Soc*. **1998**, *120*, 6745-6752.
2. Pugh, T.; Chilton, N. F.; Layfield, R. A. A Low-Symmetry Dysprosium Metallocene Single-Molecule Magnet with a High Anisotropy Barrier. *Angew. Chem. Int. Ed*. **2016**, *55*, 11082-11085.
3. Dolomanov, O. V.; Bourhis, L. J.; Gildea, R. J.; Howard, J. A. K.; Puschmann, H. OLEX2: A Complete Structure Solution, Refinement and Analysis Program. *J. Appl. Crystallogr*. **2009**, *42*, 339-341.
4. Sheldrick, G. M. Crystal Structure Refinement with SHELXL. *Acta Crystallogr. Sect. C Struct. Chem*. **2015**, *C71*, 3-8.
5. Sheldrick, G. M. SHELXT – Integrated Space-Group and Crystal-Structure Determination. *Acta Crystallogr. Sect. A Found. Adv.* **2015**, *A71*, 3-8.
6. Pugh, T.; Chilton, N. F.; Layfield, R. A. A Low-Symmetry Dysprosium Metallocene Single-Molecule Magnet with a High Anisotropy Barrier. *Angew. Chem. Int. Ed*. **2016**, *55*, 11082-11085.
7. Bain, G. A.; Berry, J. F. Diamagnetic Corrections and Pascal's Constants. *J. Chem. Educ*. **2008**, *85*, 532-536.
